# Cancer stem-like cell: a novel target for nasopharyngeal carcinoma therapy

**DOI:** 10.1186/scrt433

**Published:** 2014-03-27

**Authors:** Pingpin Wei, Man Niu, Suming Pan, Yanhong Zhou, Cijun Shuai, Jing Wang, Shuping Peng, Guiyuan Li

**Affiliations:** 1 Hunan Provincial Tumor Hospital and the Affiliated Tumor Hospital of Xiangya School of Medicine, Central South University, 582 Xianjiahu Road, Changsha, Hunan 410013, China; 2 Cancer Research Institute of Xiangya Medical School, Central South University, 110 Xiangya Road, Changsha, Hunan 410078, China; 3 Guandong Provincial Yuebei People’s Hospital, 133 Huimin South Road, Shaoguan, Guangdong 512025, China; 4 State Key Laboratory of High Performance Complex Manufacturing, Central South University, 932 South Yeulu Mountain Road, Changsha, Hunan 410083, China

## Abstract

Nasopharyngeal carcinoma (NPC) is the most common cancer originating in the nasopharynx, and is extremely common in southern regions of China. Although the standard combination of radiotherapy and chemotherapy has improved the efficiency in patients with NPC, relapse and early metastasis are still the common causes of mortality. Cancer stem-like cells (CSCs) or tumor initial cells are hypothesized to be involved in cancer metastasis and recurrence. Over the past decade, increasing numbers of studies have been carried out to identify CSCs from human NPC cells and tissues. The present paper will summarize the investigations on nasopharyngeal CSCs, including isolation, characteristics, and therapeutic approaches. Although there are still numerous challenges to translate basic research into clinical applications, understanding the molecular details of CSCs is essential for developing effective strategies to prevent the recurrence and metastasis of NPC.

## Introduction

Nasopharyngeal carcinoma (NPC) is a distinct malignancy of the nasopharynx, the uppermost region of the pharynx. NPC is highly prevalent in southern China and southeastern Asia (incidence is between 25 and 50/100,000), but is rare in the United States and most other nations (incidence < 1/100,000) [1]. More than 90% of NPC patients are initially diagnosed as type II or type III undifferentiated and nonkeratinizing carcinoma [2]. Epstein–Barr virus (EBV) infection, environment and diet, and genetic factors all contribute to the development of NPC [3].

Radiotherapy or combined chemoradiotherapy shows a cure rate >90% in patients with early-stage NPC [4], but significant rates of distant relapse and metastasis still occur in patients after radiotherapy or chemoradiotherapy. Although much progress has been gained in recent years, the 5-year survival rate is only 50 to 60% [5]. The leading cause of mortality is attributed to local recurrence and metastasis. Although numerous molecular targeting agents have been developed due to deeper understanding of the disease progression, local recurrence still occurs in 15 to 58% [6], and the rate of nasopharynx and cervical lymph node recurrence is between 12.0 and 22.0% of patients who underwent standard chemotherapy and radiotherapy treatment during 5 years [7]. Thus it is crucial to develop effective strategies to attack cancer cells that become resistant to current chemotherapy.

In recent years, one of the major mechanisms for post-therapeutic recurrence of NPC has been suggested by the cancer stem-like cell (CSC) proposition [8-10]. According to the CSC model, cancers are hierarchically organized similar to normal tissues and cancer growth and progression are driven by a small subpopulation of tumor cells with stem cell-like properties, the CSCs. This rare cell subpopulation is responsible for tumor initiation, maintenance and regeneration. CSCs have been identified in various human malignancies such as brain cancer [11], breast cancer [12], colon cancer [13,14], pancreatic cancer [15,16], and so forth.

In this paper we will summarize the studies on NPC CSCs, including isolation, characteristics, and therapeutic approaches.

## Experimental evidence for nasopharyngeal carcinoma cancer stem-like cells

Recent investigations into NPC CSCs are summarized in Table 1.

**Table 1:** Reports of expression/functional profiles recently found to identify putative human nasopharyngeal cancer stem-like cells

**Expression/functional profile**	**Cells/tissues studied**	**Characteristics of cancer stem cell identity**
Label-retaining cells	5-8 F, 6-10B and TMNE	Increased clonogenicity, tumor formation in mice at low titers [16,17]
Label-retaining cells (PKH26^+^)	CNE1, CNE2, SUNE1, and HONE1	Longevity, sphere formation, side population cells, and resistance to radiotherapy [18]
Side population cells	CNE-2	Strong tumorigenesis ability, more resistant to chemotherapy and radiotherapy, cytokine 19 positive [24]
Side population cells (ABCG2^+^)	5-8 F	ABCG2 alone is not sufficient, PSCA, ABCG2 and ALP were expressed in ABCG2^+^ cells, and K19, integrin α6, integrin β4, CD44 and K14 were expressed in ABCG2^−^ cells [25]
ALDH1^high^ cells or ALDH1A1	5-8 F and CNE2	High ALDH1 activity, higher clone formation efficiency, differentiation capability and higher migration, enhanced capacities of growth, proliferation, and tumorigenesis,5 to 10^3^ ALDH1^high^ NPC cells required to induce tumors, vimentin^+^, and E-cadherin^−^,OCT4, SOX2 and Nanog^+^[27,28]
	C666-1	Significantly greater ability to proliferate, be clonogenic, resist chemotherapy drugs and radiation, and express pluripotent markers, tumor formation at a higher rate [29-31]
CD44^+^	5-8 F, C666-1	Higher survival rate, resist chemotherapy drugs [35,36]
CD133^+^	CNE2 and primarily cultured NPC cells	Nanog^+^ and Sox2^+^, a strong potential for self-renewal, sphere formation, proliferation and differentiation and a greater potential for *in vivo* tumor formation in nude mice [39]
Sphere-forming cells	C666-1	CD44^+^ and SOX2^+^, higher spheroid formation efficiency, resistant to chemotherapeutic agents, CCR7^+^ associated recurrent disease and distant metastasis [48]

*ABCG2* ATP-binding cassette sub-family G member 2; *ALDH1* aldehyde dehydrogenase 1; *NPC* nasopharyngeal carcinoma.

### Label-retaining cells

Adult stem cells can be identified by the label-retaining cell (LRC) approach based on their ability to retain nucleoside analogs, such as bromodeoxyuridine. Zhang and colleagues identified LRCs from nasopharyngeal epithelia when neonatal mice were intraperitoneally injected with bromodeoxyuridine [17]. Long-term bromodeoxyuridine-labeled LRCs (2% of cells) were detected in the adult mice nasopharyngeal epithelia by immunostaining, and some LRCs (12% of cells) were found to be recruited into the S phase of the cell cycle with an additional radioactive thymidine-labeling technique, indicating that the stem cells also divide, most probably asymmetrically. In addition, three NPC cell lines (5-8 F, 6-10B and TMNE) were labeled with bromodeoxyuridine *in vitro* and then individually engrafted into the back of nude mice, where the tumors develop. Label-retaining stem cells were found in all three kinds of NPC xenograft tumors (0.3% of cells), around 16% of which were also labeled with radioactive thymidine. The presence of epithelial LRCs in mouse nasopharynx and human NPC tissues was demonstrated, and these stem-like LRCs are not completely quiescent as they can be recruited into the cell cycle to participate in the physiological or pathological process at any moment. Jiang and Yao also verified LRCs existing in NPC cell line 5-8 F [18]. Bromodeoxyuridine was detected in 5-8 F cells and xenograft tumors. After 8 weeks, only sporadic LRCs were detected in xenograft tumors, and these LRCs were located at the cancer margin. The existence of LRCs in 5-8 F cells indicates the existence of cancer stem cells in NPC.

PKH26 is a lipophilic marker that intercalates into the membranes of viable cells and is not transferred between cells. A stem cell-like subpopulation (PKH26^+^) was identified in NPC cell lines using a label-retention technique [19]. PKH26^+^ cells were enriched for clonogenicity, sphere formation, side population (SP) cells, and resistance to radiotherapy. They also found that proto-oncogene c-MYC regulates radiotolerance through transcriptional activation of CHK1 and CHK2 checkpoint kinases by binding to the promoters using genomic approaches. Overexpression of c-MYC in the PKH26^+^ subpopulation leads to increased expression of CHK1 and CHK2 and subsequent activation of the DNA damage-checkpoint response, resulting in radioresistance. Furthermore, loss of CHK1 and CHK2 expression reverses radioresistance in PKH26^+^ cells *in vitro* and *in vivo*. This study elucidates the role of the c-MYC-CHK1/CHK2 axis in regulating DNA damage-checkpoint responses and stem cell characteristics in the PKH26^+^ subpopulation. Furthermore, these data reveal a potential therapeutic application in NPC.

### Side population cells

SP cells in tumors are a small subpopulation of cancer cells with stem-like properties that can be identified by flow cytometry analysis based on their high ability to export certain compounds such as Hoechst 33342 and chemotherapeutic agents. The existence of SP cells in tumors is considered a key factor contributing to drug resistance and metastasis [20-24], and presents a major challenge in cancer treatment. SP cells have been isolated from several solid tumors. NPC is characterized by lymph node of the neck and cranial nerve involvement at the early stage when the primary tumor is small or not obvious. Wang and colleagues isolated SP cells from five NPC cell lines and investigated stem cell characteristics, such as proliferation, self-renewal, and differentiation [25]. They observed a strong tumorigenic ability of SP cells following *in vivo* transplantation into immunodeficient mice. SP cells were found to be more resistant to chemotherapy and radiotherapy, and this was related to the ATP-binding cassette half-transporter member 2 of G family protein and smoothened protein expression, respectively. These data showed that SP cells in human NPC cell line CNE-2 had stem cell characteristics *in vitro* but also showed that they had a strong ability to form tumors *in vivo*.

ATP-binding cassette sub-family G member 2 (ABCG2) transports various molecules across extracellular and intracellular membranes. High ABCG2 expression subpopulation cancer cells are associated with multidrug resistance. The SP phenotype is mainly mediated by ABCG2. Zhang and colleagues isolated ABCG2^+^ cells in 5-8 F NPC cells and compared their tumorigenic potential with ABCG2^−^ cells using the magnetic cell sorting method [26]. Cell cycle analysis indicated that ABCG2^+^ cells were mostly quiescent, and they showed lower cloning efficiency and tumorigenicity than ABCG2^−^ cells. Using the Affymetrix human whole genome expression chip to identify the gene expression profile of ABCG2^+^ and ABCG2^−^ cells, both subpopulations expressed some stem cell-associated genes; for example, PSCA, ABCG2 and ALP were expressed in ABCG2^+^ cells, and K19, integrin α6, integrin β4, CD44 and K14 were expressed in ABCG2^−^ cells, suggesting there were stem cells in both ABCG2^+^ and ABCG2^−^ cells. This observation demonstrated that there exist ABCG2^+^ cells in NPC cells, but ABCG2 alone is not sufficient for isolating cancer stem cells in 5-8 F NPC cells. Similar results have been obtained in other tumors [27]. This evidence indicates that ABCG2 alone should not be a reliable stem cell marker.

### ALDH1^high^ cells

Aldehyde dehydrogenase 1 (ALDH1) belongs to the aldehyde dehydrogenase superfamily, which is responsible for the oxidation of aldehydes to their corresponding carboxylic acids. ALDH1 was first used as a marker of cancer stem cells in hematopoietic cells [28]. Wu and colleagues isolated ALDH1^+^ cells from human NPC cell lines (5-8 F and CNE2) and found that ALDH1^+^ cancer cells grew faster and had higher clone formation efficiency, differentiation capability and higher migration than ALDH1^−^ cancer cells *in vitro*[29]. In addition, ALDH1^+^ cancer cells formed significantly more tumor spheres. *In vivo* experiments showed that only 5 to 10^3^ ALDH1^+^ NPC cells were required to induce tumors. Notably, ALDH1^+^ cells were enriched in the SP and stem cell-like genes OCT4, BMI1, KLF4 and SOX2 are preferentially expressed in ALDH1^+^ cells. The expression of ALDH1 correlated significantly with TNM classification and epithelial–mesenchymal transition (EMT) markers including vimentin expression and loss of E-cadherin.

ALDH1 might be a novel stem cell marker and a valuable predictor of NPC with poor survival [30]. Yu and colleagues showed that enriched CSCs with high ALDH activity in C666-1 were associated with a significantly greater ability to proliferate, be clonogenic, resist chemotherapy drugs and radiation, reconstitute a heterogeneous population, and express pluripotent markers [31]. Two groups have investigated the expression of ALDH1A1 in NPC specimens [32,33]. Their findings indicated that increased expression of ALDH1A1 in NPC was associated with stem-like properties.

### CD44^+^ cells

CD44 is a cell-surface glycoprotein involved in cell–cell interactions, cell adhesion and migration. CD44 cells are reported as cell surface markers for some breast and prostate cancer stem cells [34,35]. Su and colleagues isolated CD44^+^ cells from NPC cell line 5-8 F by fluorescence-activated cell sorting [36]. CD44 was positively expressed in 52.5% of the 5-8 F cell line. Regardless of the culture conditions, with or without serum, freshly sorted CD44 cells showed stronger proliferative capacity than CD44^−^ and unsorted cells. The expression levels of B-lymphoma Mo-MLV insertion region 1 homolog (Bmi-1) and Oct-4 mRNA in CD44^+^ cells were significantly higher than for CD44^−^ cells. This study indicated that CD44^+^ cells have the biological characteristics of CSCs or cancer progenitor cells (CPCs): pluripotent marker expression, high self-renewal ability, and chemoresistance and radioresistance. Janisiewicz and colleagues reported that CD44^+^ cells differentiated into CD44^−^ cells in C666 cells [37]. CD44^+^ cells exhibited tumor-initiating capacity in the xenograft model. Patient tumors were heterogeneous for CD44 staining, and CD44 expression is associated with clinical outcome. These observations indicated that the CD44^+^ subpopulation has features consistent with CSCs.

The expression of CD44 and its role as a marker of cancer stem cells in the head and neck is interesting. Recent publications have pointed out the role of CD44 as a CSC marker in the early diagnosis of head and neck squamous cell cancer and as a prognostic factor for relapse, recurrence of disease and chemo/radioresistance [38,39]. As this point, CD44 is correlated with the outcome and prognosis in head and neck squamous cell cancer and is associated with cancer stem cells if combined with other surface markers of cancer stem cells.

### CD133^+^ cells

CD133 is a member of pentaspan transmembrane glycoproteins (5-transmembrane), which specifically localize to cellular protrusions. CD133 has been found to be expressed in hematopoietic stem cells, endothelial progenitor cells, glioblastoma, neuronal and glial stem cells and various pediatric brain tumors. A CD133^+^ cell population was enriched using magnetic-activated cell sorting technology in NPC cell line CNE2 by Wen’s group [40]. CD133^+^ cells exhibited a stronger potential for self-renewal, proliferation and differentiation and a greater potential for *in vivo* tumor formation in nude mice compared with CD133^−^ cells, although the percentage of CD133^+^ cells was small. These results suggest that CD133 may serve as a specific surface marker for nasopharyngeal cancer stem cells.

### Other factors

Bmi-1 has been reported as an oncogene by regulating p16 and p19, which are cell cycle inhibitor genes. Bmi-1 is necessary for efficient self-renewing cell divisions of adult hematopoietic stem cells as well as adult peripheral and central nervous system neural stem cells. Overexpression of Bmi-1 has been found in various malignant tumors as well as in NPC [41]. Upregulation of Bmi-1 induced EMT and enhanced the motility and invasiveness of human nasopharyngeal epithelial cells, while Bmi-1 depletion enhanced the chemosensitivity of NPC cells by inducing apoptosis. Bmi-1 was therefore assumed to be a candidate cancer stem cell marker [36]. In the literature there are reports that corroborate the role of Bmi-1 as a prognostic factor for local recurrence and distant metastases in laryngeal carcinoma [42,43].

### Sphere-forming cells

The sphere culture assay has been used to enrich the potential CSC subpopulations when the specific CSC makers have not been defined, as is the case for most CSCs [44-48]. The character relies on their property of anchorage-independent growth in serum-free medium. Lun and colleagues reported that sphere-forming cells were isolated from an EBV-positive NPC cell line C666-1 and its tumor-initiating properties were confirmed [49]. Both CD44 and SOX2 were found to be overexpressed in a majority of sphere-forming C666-1 cells. The CD44^+^SOX2^+^ cells were detected in a minor population in EBV-positive xenografts and primary tumors and were considered as potential CSCs in NPC. Notably, the isolated CD44^+^SOX2^+^ NPC cells were resistant to chemotherapeutic agents and had higher spheroid formation efficiency, showing CSC properties.

## Model on origin of nasopharyngeal carcinoma cancer stem-like cells

**Figure 1:**
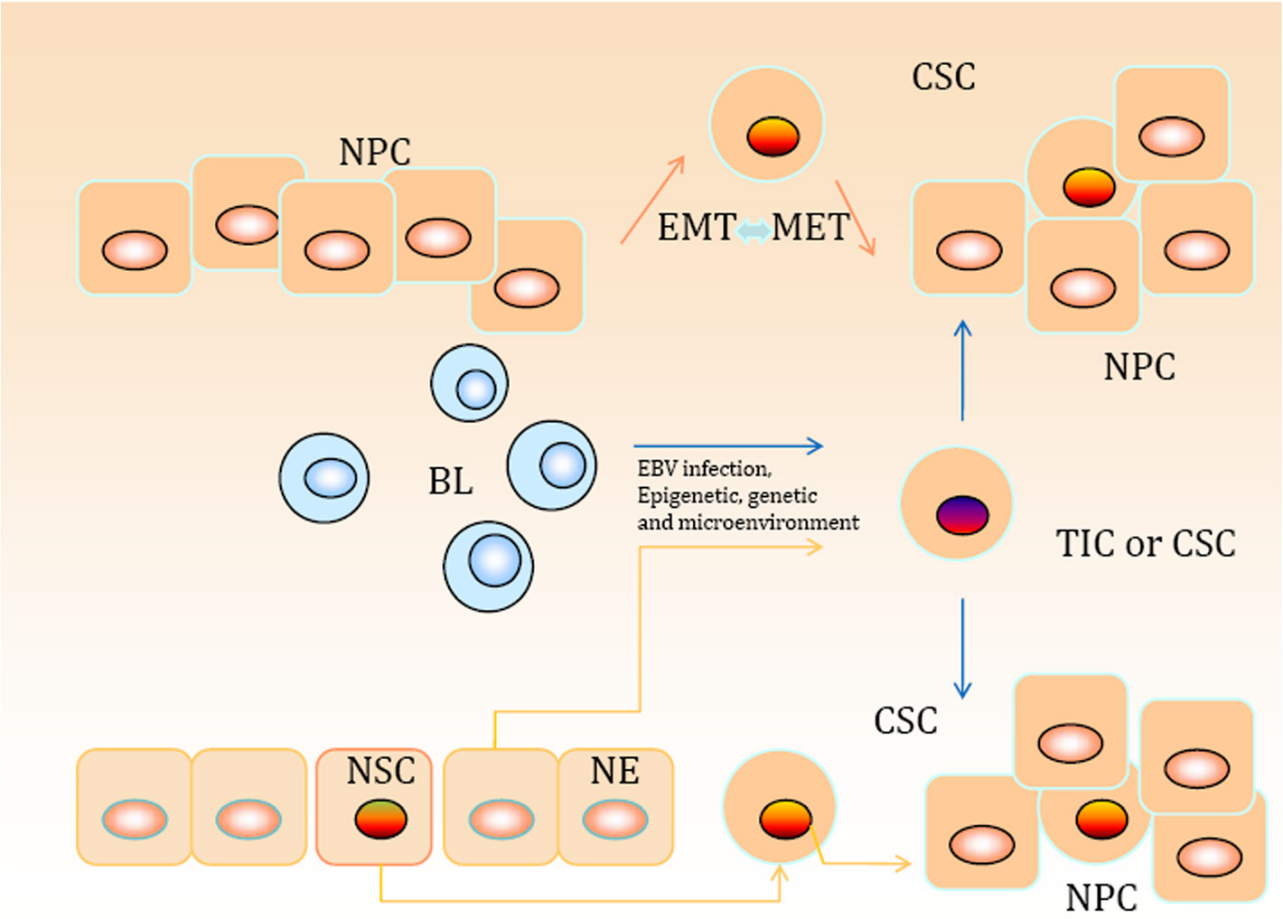
**Model for origin of nasopharyngeal carcinoma stem or stem-like cells.** BL, B lymphocytes; CSC, cancer stem-like cell; EBV, Epstein–Barr virus; EMT, epithelial–mesenchymal transition; MET, mesenchymal–epithelial transition; NE, nasopharyngeal epithelial cells; NPC, nasopharyngeal carcinoma; NSC, normal stem cell; TIC, tumor-initiating cell.

The origin of CSCs is still controversial. Several hypotheses indicate that their origin may be heterogenic (Figure 1).

### Loss of differentiation of nasopharyngeal mucosa B lymphocytes or epithelial cells

NPC is predominantly undifferentiated and nonkeratinizing. The vast majority of cases of this cancer reveal not only varying degrees of squamous differentiation, but also more or less preserved traces of the columnar cell differentiation under electron microscopy. In other words, NPC is a kind of biphasic malignant tumor with squamous differentiation with a slight advantage [50]. EBV genome analysis in the tumor cells shows that only one fusion terminal fragment exists, suggesting that viral DNA of all NPC cells is homologous and originates from the same clone proliferation [51]. Viral DNA present in the EBV-infected lymphocytes infiltrated inside the tumor can be easily traced by polymerase chain reaction amplification [52,53]. One may therefore suggest a hypothesis: B lymphocytes are not terminally differentiated cells or the B-cell terminal differentiation capacity can be reversed by the role of a certain factor, and then by the nasopharyngeal mucosa. B lymphocytes may be the progenitor cells of NPC. However, there is still no evidence to support this hypothesis.

EBV-encoded small RNA signals were also recently detected in the nuclei of NPC cells, and occasionally in a limited number of infiltrating lymphocytes by quantum-dot fluorescent *in situ* hybridization [54]. In fact, the nasopharyngeal epithelial cells with some prior genetic changes are prone to transformation, and then premalignant lesions and the concurrent detection of dysplasia and invasive carcinoma of epithelial cells occur. The viral episome is maintained in the infected epithelial cell, which continues to proliferate and does not differentiate [50]. This means that the transformation of nasopharyngeal epithelial cells with genetic modifications attacked by EBV may also be the origin of NPC CSCs.

### Epithelial–mesenchymal transition induces cancer stem/progenitor-like cells

Evidence has been found that the principal oncoprotein of EBV, latent membrane protein 1 (LMP1) [55] – which is associated with human malignancies, especially NPC – promotes tumor cell invasion and metastasis, as well as the EMT. Horikawa and colleagues [55] reported that LMP1 induces the CD44^high^CD24^low^ CSC/CPC-like phenotype as well as self-renewal abilities in LMP1-expressing epithelial NPC cell lines. LMP1 increased the expression of several CPC markers as well as producing increased levels of EMT markers. These findings indicate that LMP1 can induce CPC-like properties in epithelial cells and suggest that LMP1-induced phenotypic changes contribute to the development of NPC. At the apex of the hierarchy are primitive rare CSCs, which possess extended self-renewal capabilities that allow the cells to perpetuate themselves and develop into CPCs. These CPCs have only limited self-renewal abilities and can, in turn, differentiate into various types of cancer cells. *In vivo*, the primitive rare CSCs rarely divide, whereas CPCs proliferate rapidly. In addition, latent membrane protein 2a also has similar roles in inducing EMT [56,57].

Induction of EMT in tumor cells not only promotes tumor cell invasion and metastasis, but also contributes to drug resistance [25,58-61]. These processes are consistent with the acquisition of a CSC phenotype that is also known as stemness characteristics [62]. Although CSCs may form in such a way, EMT acts as part of the process and mesenchymal cells partly have the properties of cancer stem cells.

### Originating from normal stem cells

CSCs share similar properties with normal stem cells, such as a long lifespan, induction of angiogenesis, resistance to apoptosis, ability for self-renewal and differentiation, expression of stem cell markers, and so forth [46]. Normal nasopharyngeal stem reserve cells of columnar epithelium and the basal cells of squamous epithelium stem cells, especially basal cell foci of squamous metaplasia, have been considered the origin of NPC cells. Zhang and coworkers first described the identification of stem-like cells in normal mouse nasopharyngeal epithelium with the well-established LRC approach [17]. EBV infection, environment and diet, and genetic factors can promote the transformation and abnormal differentiation of normal stem cells, which may be a derivative of NPC CSCs.

## Therapeutic approaches targeting nasopharyngeal cancer stem-like cells

**Figure 2:**
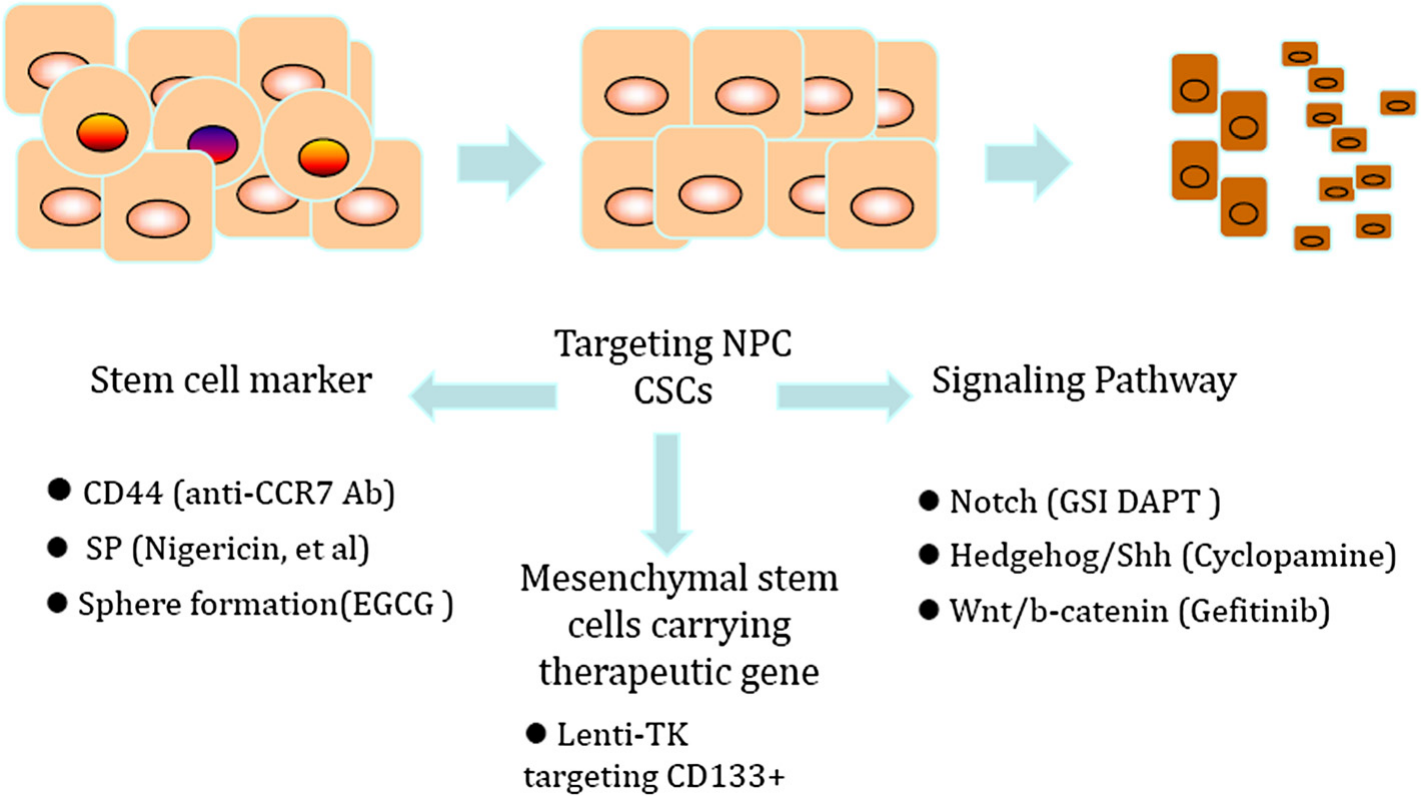
**Potential therapeutic approaches targeting nasopharyngeal carcinoma stem cells or stem-like cells.** Ab, antibody; CCR7, CC-chemokine receptor 7; CPC, cancer progenitor cell; DAPT, (*N*-((3,5-difluorophenyl)acetyl)-l-alanyl-2-phenyl)glycine-1,1-dimethylethyl ester; EGCG, epigallocathechin gallate; GSI, inhibitor of γ-secretase; NPC, nasopharyngeal carcinoma; SP, side population; TK, tyrosine kinase.

Ongoing studies show that targeting CSCs may be a quite promising strategy. Based on the properties of NPC CSCs, many attempts have been developed to target NPC CSCs specifically (Figure 2).

Nigericin was recently reported to target CSCs selectively and to sensitize CSCs in NPC to the widely used clinical drug cisplatin both *in vitro* and *in vivo*. Nigericin decreased the SP percentages in S18 cells (higher metastatic ability) and S26 cells (much lower metastatic ability) derived from the NPC cell line CNE-2. The downregulation of the polycomb group protein Bmi-1 was found to contribute to the inhibitory effect of nigericin on CSCs [41]. Blocking of CCR7 with anti-CCR7 antibody abolished the sphere-forming ability of C666-1 *in vitro*[49].

Shen and colleagues found that resveratrol impeded CSC properties through the activation of p53 and this effect could be reversed by knockdown of p53. Furthermore, resveratrol suppressed the stemness and EMT through reactivating p53 and inducing miR-145 and miR-200c, which were downregulated in NPC CSCs [63]. Epigallocathechin gallate, the most abundant catechin in green tea, has been reported to regulate NPC CSCs and their self-renewal capacity, and inhibited their invasive characteristics [64]. Smac mimetics in combination with TRAIL selectively target cancer stem cells, reduce the percentage of SP cells, inhibit colony-forming and sphere-forming abilities, and eliminate NPC stem cells in xenograft mice [65].

Notch signaling is important for the self-renewal and maintenance of stem cells. In cancer stem cells, the signaling pathway commonly is activated. Yu and colleagues reported that the Notch inhibitor, (*N*-((3,5-difluorophenyl)acetyl)-l-alanyl-2-phenyl)glycine-1,1-dimethylethyl ester, could reduce the proportion of SP cells from the NPC cell line CNE1,2 [66] . This ester inhibited NPC cell proliferation, depleted SP cells, reduced colony formation, and tumor formation of xenograft in immune-deficient nude mice, and induced apoptosis of NPC cells. This study shows that Notch pathway inhibition may be a promising clinical approach in CSC-targeting therapy for NPC.

The epidermal growth factor receptor pathway plays a critical role in regulating CSCs [67]. The effects of epidermal growth factor receptor on maintaining CSCs are mainly mediated by AKT signaling, and β-catenin is responsible for governing CSC properties in response to epidermal growth factor receptor/AKT activation. Tumor cells derived from cisplatin-treated mice grew rapidly, whereas regrowth of tumor cells from gefitinib-treated mice was severely diminished. Expression of epidermal growth factor receptor correlates with expression of β-catenin and nanog in primary tumor specimens from NPC patients. These findings provide mechanistic and preclinical evidence supporting the use of gefitinib alone or in combination with a chemotherapeutic agent in the therapy for patients with NPC. Targeting β-catenin is suggested to represent a rational clinical strategy for CSCs harboring activated epidermal growth factor receptor or AKT.

The hedgehog pathway, a pathway implicated in the maintenance of stem cells, is activated by the binding of a hedgehog ligand (Sonic hedgehog) to the transmembrane protein receptor Patched, releasing the inhibition of Smoothened and then activating downstream signaling components and Gli-mediated transcription of target genes. EBV activates the hedgehog signaling pathway through autocrine induction of Sonic hedgehog ligand [68,69]. Blocking the pathway with cyclopamine, a specific inhibitor of the Sonic hedgehog signaling pathway, reduced the proliferation of NPC epithelia cell lines and induced the apoptosis of NPC cells.

## Conclusion and future perspectives

From the biological behavior of the EBV and nasopharyngeal cancer-specific cell surface markers, we hypothesized that the generation of NPC may be derived from undifferentiated clonal expansion of B lymphocytes or dedifferentiation of epithelial nasopharynx by EMT reprogramming or mutation of normal nasopharyngeal epithelial cells. These assumptions still require further experimental evidence. Similar to the heterogeneous nature of solid cancers, the origin of cancer stem cells in different individuals with the same type may vary due to epigenetic, genetic and tumor microenvironment factors.

Although many of the studies showed that these compounds have considerable promise, few provided comprehensive evidence showing that the proposed agents were specific to NPC CSCs and were considerably more effective than conventional therapy (radiation, cisplatin, and so forth). These proposed treatments still require further investigation, especially through rigorous *in vivo* experimentation, before they can be considered true potential CSC inhibitors and considered for use in clinical trials.

The investigations into the specific NPC CSCs and clinical therapeutic trials have been ongoing. However, there remains much to do in improving the therapeutic target specific for NPC CSCs. Several factors should be considered; first, the reliability of using cell surface markers or functional screening as a method to isolate CSCs remains controversial. Golebiewska and colleagues have reported that SP cells in human glioblastoma are non-neoplastic and are exclusively stroma derived [70]. ABCG2^+^ and ALDH1^high^ cannot act alone as cancer stem cells biomarkers [26,71,72]. A second factor is that the heterogeneous nature of NPC cells and NPC CSCs requires obtaining an individualized treatment plan. More investigations are therefore required to acquire the primary characteristics and the molecular regulation expression profile in cancer stem cells.

CSCs from various tissues appear to be more resistant to chemotherapeutic reagents than do mature cell types and characteristically express drug-resistance proteins. If this was true of NPC CSCs, therapies targeting CSCs directly should result in more reliable responses for primary as well as metastatic disease. The sensitivity of CSCs to the different strategies requires more experimental evidence confirmed both *in vitro* and *in vivo*. With these targets known, this fraction of cancer cells that can rapidly develop the critical tumor cell mass can be eliminated (Figure 2). Consequently, defining the unique properties of NPC CSCs remains a high priority for developing early diagnostic and effective therapeutic strategies against NPC.

## Abbreviations

ABCG2: ATP-binding cassette sub-family G member 2; ALDH1: Aldehyde dehydrogenase 1; Bmi-1: B-lymphoma Mo-MLV insertion region 1 homolog; CPC: Cancer progenitor cell; CSC: Cancer stem-like cell; EBV: Epstein–Barr virus; EMT: Epithelial–mesenchymal transition; LMP1: Latent membrane protein 1; LRC: Label-retaining cell; NPC: Nasopharyngeal carcinoma; SP: Side population.

## Competing interests

The authors declare that they have no competing interests.
